# Words Are Essential, but Underexamined, Research Tools for Microbes and Microbiomes

**DOI:** 10.1128/msystems.00769-21

**Published:** 2021-08-31

**Authors:** Erika Szymanski

**Affiliations:** a Department of English, Colorado State University, Fort Collins, Colorado, USA

**Keywords:** discourse, engineering, metaphor, microbiome, science and technology studies, synthetic biology, synthetic yeast

## Abstract

Language constitutes an essential set of scientific construction tools, not only for communicating knowledge, but for conceptualizing the world. Metaphors in particular, as conventions that guide and reproduce analogical reasoning, merit attention that they largely do not receive. My research addresses this deficit by examining how metaphors for handling microbes shape possibilities for working with yeast and bacteria in synthetic biology, microbiome research, and other fields that reconfigure what microbes can be. Though poised to reexamine assumptions, these fields routinely rest on metaphors and other language tools that quietly embed ways of thinking that may work against wider aims—for example, imagining bacteria as imperfect machines that should therefore be rendered increasingly passive and controllable. Researchers, therefore, need to examine how language tools structure their observations and expectations so that the tools they choose are appropriate for the work they want to do.

## COMMENTARY

Researchers are accustomed to thinking about how experimental equipment and techniques shape the data that they can and cannot collect. We less routinely think about language in the same way. Yet, discourse, or language as a social practice, is equally important to constructing scientific knowledge, from the questions that we can imagine to what we see in our data to how we communicate our findings. My research investigates how metaphors and other language used to handle microbes shape what we know about and do with them and possible trajectories for how microbes and humans may continue to work together.

When scientists do not examine what language choices do, including the assumptions they (often invisibly) carry, conceptual innovation can be limited in fields otherwise poised to innovate. For example, in a recent collaboration with a computational biologist, we observed that every computer-aided DNA design tool in current use is grounded in the same understanding of the genetic code as a context-free grammar (1). All assume that functional genetic units are linear segments of text with discrete beginnings and ends, equivalent to words in the “book of life.” Furthermore, these programs assume that book to be written in a programming-like language, in which each word is associated with exactly one meaning independent of context—very much unlike human languages, but consistent with how Tom Knight, a computer scientist, expected DNA to work when he jumpstarted synthetic biology by analogizing genes to computer circuitry (2). While this approach has enabled substantial achievements in DNA design, its inability to accommodate much of the extratextual and contextual complexity of genetic function means that *in silico* designs routinely fail to function as expected *in vivo* (3). It goes without saying that incredible work has been done on the back of “the genetic code,” but science that expands this metaphor into increasingly new territory is also now colliding against limitations baked into the metaphor itself.

## METAPHORS ARE INTRINSIC TO SCIENCE

**FIG 1:**
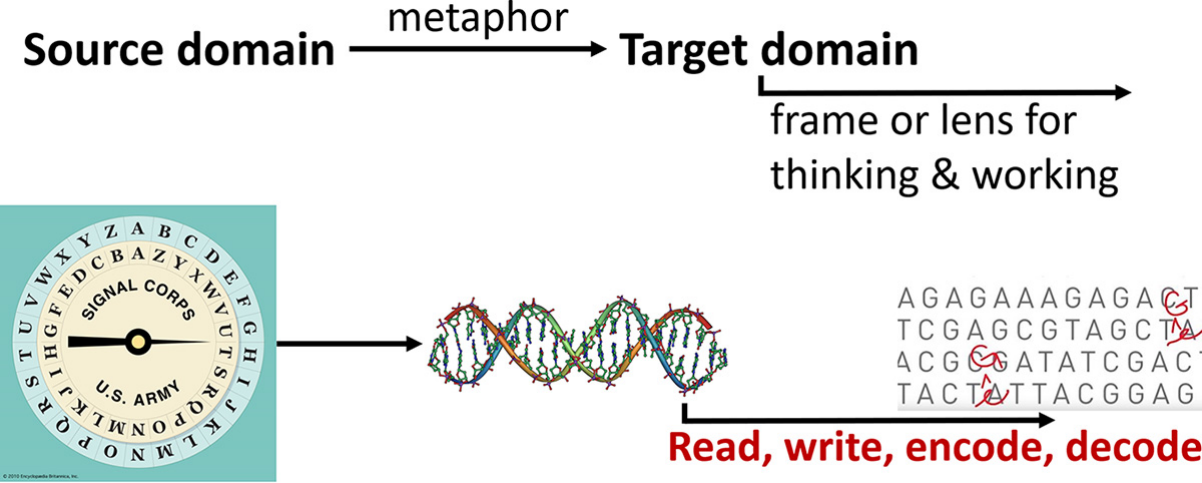
Structure of metaphors as it applies, for example, to the genetic code.

While “metaphor” may bring to mind poetic or figurative language, all language is metaphorical in the sense that we must constantly make sense of new observations and experiences in terms of previous ones (Fig. 1). Operational metaphors, such as the genetic code, mobilize sufficient conceptual and physical resources to be reinforced by the work done through them so that observations that fit the metaphor become easier to perceive and manipulate, while those not encompassed by the metaphor become easier to systematically ignore (4, 5). These are therefore tools not only for understanding the world but for remaking it, as we reconstruct objects through the tools we use to handle them. We now recode and decode, edit and rewrite, debug and refactor DNA. Of course, not all metaphors are equally useful tools; superficial metaphors such as “Juliet is the sun” cohere with little else. But operational metaphors, unavoidable in ordinary and scientific language, become so intrinsic to ways of working that we often stop seeing them as metaphors at all. Every time we use them, they reproduce the assumptions they carry and we extend the territory they structure.

## METAPHORS CONFIGURE WAYS OF WORKING WITH MICROBES

Metaphors have been widely investigated in public understanding of science, especially around genomics and synthetic biology (6–8). However, metaphors in disciplinary scientific language, in their role as part of the toolkit for doing research, have attracted far less attention. My work addresses this gap for language in microbial synthetic biology and engineering microbiomes. I have observed that while machine and programming metaphors are widespread, they come with limitations because they do not encompass much of the activity of the microbe; indeed, they tend to make the activity of the microbe a problem to be erased (9, 10). As the primary referent for “the genetic code” has slipped from cryptography to computing, cells have been seen as not only machines but machines operated by genomes analogized to cellular operating systems. Cells themselves are routinely described as “chassis,” analogous to the metal frame of an automobile onto which custom parts can be loaded, sometimes even without seeing these expressions in light of their underpinning machine metaphors. Through them, the cell becomes a container for engineered genetic pathways and circuits, running genetic programs but otherwise staying out of the way. Microbial responses to genetic constructs become “bugs” that interrupt efforts to reprogram them. While individual scientists often speak informally about the liveliness of their microbial research partners, little of this perspective seeps into publications or tool-development to become part of the established scientific infrastructure (10).

**FIG 2:**
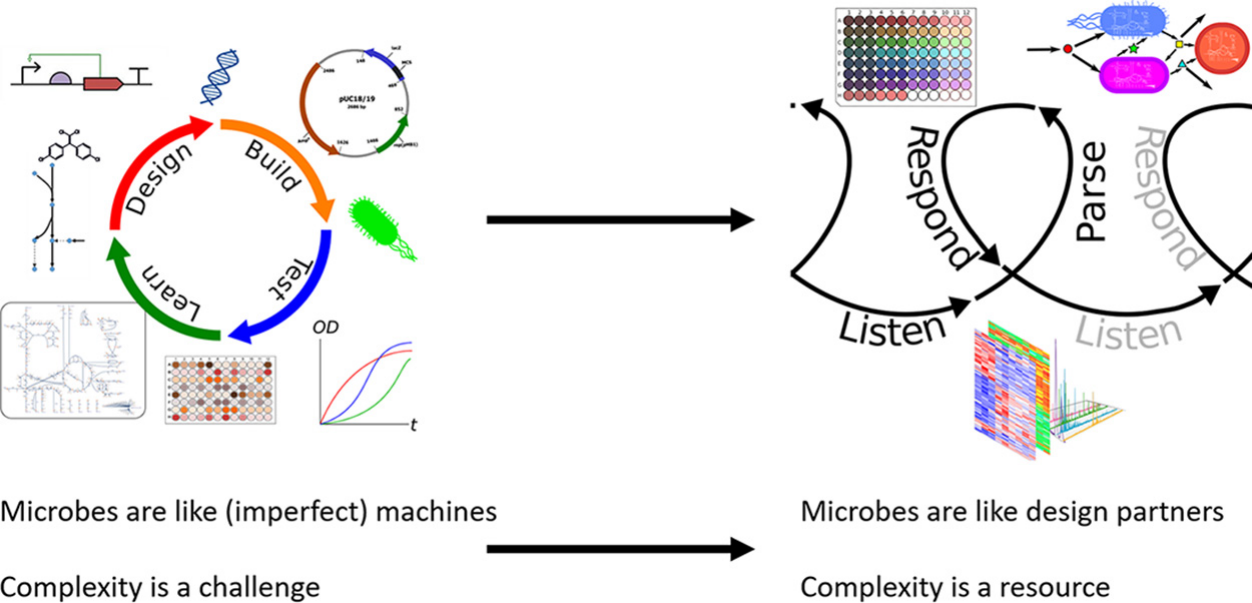
Reframing biological complexity as a resource, not a challenge, by reimagining relationships with microbial communities.

Much of microbes’ utility in engineering biology stems from their complex responsiveness; this field is exciting precisely because machine metaphors are imperfect. But, because their complexity interferes with predictable, machine-like structure-function modularity, complexity is widely described as engineering biology’s greatest challenge (11). Working with microbes as creatures that might make unique creative contributions to multispecies projects—more like design partners, less like metal frames (12, 13)—invites strategies for productively employing more of their activity rather than working to shut it down, akin to some strategies already suggested via engineering with evolution (14, 15). Through such a shift, microbial complexity might be seen as engineering biology’s greatest resource rather than its greatest challenge (Fig. 2).

The NSF-funded CAREER project that I begin this fall will test how alternative metaphors may afford diverse experimental possibilities for mechanistic microbiome research, with particular attention to foregrounding processes and relationships over objects. Attending to how metaphors enable or disenable particular forms of work is especially important in developing interdisciplinary fields wherein conventions have often not yet been codified. While microbiome research is consolidating around dominant approaches, it still remains heterogenous, with space for diverse disciplinary influences and conceptual innovation (16). This is also an important time to investigate microbiome discourse because the field is shifting from a phase of primarily correlational studies toward seeking to describe and manipulate mechanisms underlying those correlations. The tools that enabled the first phase of the field may not be sufficient or advantageous for its second phase, especially in terms of capturing relational changes over time (17).

Part of the impetus for my work comes from observing that microbiome research largely recapitulates cell- and species-centric assumptions, embedded in much of our language for handling microbes, but that represent carryovers from a biology that largely predates genomics (17). These make phenomena such as horizontal gene transfer and open pangenomes exceptions to general rules, even as such mechanisms appear to be routine in microbiomes. Naturalizing these assumptions in language for doing microbiome research has consequences for, among other things, how sequencing data are bucketed and how a valid sequence is distinguished from noise. Because microbiome research tends to address communities over individuals, it has tremendous promise to revisit propositions that continue to be reproduced even as they make sense of a decreasing share of what we know about microbial life, such as each cell possessing exactly one genome (18). However, the field needs to employ the right tools for that job.

Preliminary studies indicate that less-common approaches in microbiome research center metabolic processes over cellular compartments, sometimes resembling social science and humanities theories that advocate centering processes over objects. I will therefore explore the potential productivity of bringing microbiome research together with relational, process-focused, and verb-focused approaches from history and philosophy of science and feminist science and technology studies (5, 19–21). Researchers in these fields have advanced alternatives to “object-based mastery” as ways to foreground the importance of relationships in how humans and other creatures develop with each other, rather than foregrounding the set of relationships described under “control.” (22) Where microbiome research may help test what social science and humanities theories do in practical application, social science and humanities theories may inspire new approaches in microbiome research.

## LANGUAGE CHOICES ARE SIMULTANEOUSLY SCIENTIFIC AND POLITICAL

Scientific discourse research is compelling for both scientific and political reasons because questions about language are simultaneously epistemological questions and political questions (23, 24). Rhetoric of science has at times seemed like an odd idea because—so the story went—if science is a matter of uncovering universal truths about the world, then scientific language simply needs to be as neutral and transparent as possible so as to transmit those facts without interference. However, that way of thinking requires imagining that scientific knowledge is unaffected by context, a view which finds little support in today’s diverse societies or in the historical record. The “war on COVID-19” comes to mind, as does control and communication language in molecular biology as shaped by early twentieth century American and British military interests in cybernetics (25). Because knowledge is made and not simply found, language is an inescapable knowledge construction tool (26). Language involves choices, and choices involve values. Because we have choices about which tools to employ, we are never only asking, “what characterizations of biology are most accurate?” We are also simultaneously asking, “what characterizations of biology best serve our goals?” and “what kinds of worlds do we *want* to build?”

Researchers have both a scientific and a societal responsibility to ensure that their research tools enable the kinds of futures that they want to build (27). Much of biology discourse has its roots in an era that was unapologetically anthropocentric and concerned with the wellbeing of only a limited number of humans. Today, bits of biological discourse are easily shaped by specific capitalist interests, yielding tools that may not necessarily be apt for enabling sustainable futures in which diverse humans and other creatures flourish together. Some of these choices have had profound, systematic, and damaging consequences for life on earth. Everyone who has a stake in how humans continue to work with microbes may not want to make the same choices, but we all need to see that there are choices to be made.
